# Fetuin, Matrix-Gla Protein and Osteopontin in Calcification of Renal Allografts

**DOI:** 10.1371/journal.pone.0052039

**Published:** 2012-12-17

**Authors:** Johan M. Lorenzen, Filippo Martino, Irina Scheffner, Verena Bröcker, Holger Leitolf, Hermann Haller, Wilfried Gwinner

**Affiliations:** 1 Department of Medicine, Division of Nephrology & Hypertension, Hanover Medical School, Hannover, Germany; 2 Department of Pathology, Hanover Medical School, Hannover, Germany; 3 Department of Medicine, Division of Endocrinology, Hanover Medical School, Hannover, Germany; 4 Institute of Molecular and Translational Therapeutic Strategies (IMTTS), Hanover Medical School, Hannover, Germany; The University of Manchester, United Kingdom

## Abstract

**Background:**

Calcification of renal allografts is common in the first year after transplantation and is related to hyperparathyroidism. It is associated with an impaired long-term function of the graft (Am J Transplant 5∶1934-41, 2005). Aim of this study is to examine the role of the anti-calcifying/calcifying factors in the pathophysiology of renal allograft calcification.

**Methods:**

We analyzed protocol allograft biopsies, blood and urine samples of 31 patients with and 27 patients without allograft calcification taken at 6 weeks, 3 and 6 months after transplantation. Patient demographical data, cold ischemia time, initial graft function and donor characteristics were comparable between the two groups. Biopsies were stained for osteopontin, fetuin, and matrix-gla-protein. Serum and urine electrolytes, matrix-gla-protein, fetuin, Vitamin D and intact parathyroid hormone in serum and osteopontin (OPN) in urine were examined.

**Results:**

Serum levels of fetuin and matrix-Gla protein as well as urinary levels of OPN showed specific time dependent changes (6 weeks vs. 3 months vs. 6 months; all p<0.0001). In patients with calcifications, urinary levels of OPN were decreased by 55% at 6 weeks and increased thereafter, showing 54% higher levels at 6 months compared to patients without calcification (6 weeks: p<0.01, 6 months: p<0.05). Local protein expression of fetuin-A, matrix-Gla protein and OPN in the graft was specifically increased around calcified plaques, without differences in the mRNA tissue expression.

**Conclusion:**

Anticalcifying factors show significant changes in the early phase after renal transplantation, which may be important for the prevention of allograft calcification. The differences in OPN indicate an involvement of this factor in the regulation of calcification.

## Introduction

The introduction of potent immunosuppressive drugs in the past three decades was the basis for a dramatic reduction of early graft loss in kidney transplant recipients. However, with respect to long-term graft survival improvements were only modest in nature [1]. Chronic allograft dysfunction is mainly related to ischemia-reperfusion injury, insufficiently controlled acute and chronic rejection and calcineurin-inhibitor toxicity [2]. Recently, calcification of renal allografts has been identified as an additional pathophysiological factor in graft deterioration. The prevalence of tubular microcalcification was shown to reach 42.7% one year after transplantation and 78.5% ten years after transplantation [3]. The prevalence of early calcifications was 6.1% at six weeks and increased to 17.8% at six months after transplantation and this was associated with an inferior long-term graft function. Calcifications were shown to be related to hyperparathyroidism and higher serum calcium levels [4]. However, a considerable overlap of calcium and parathyroid hormone levels between patients with and without calcification was observed, indicating that other factors must be important for the development of calcifications. Multiple soluble, circulating factors and local tissue factors are presumed to regulate tissue calcification by inducing or inhibiting the deposition of calcium-containing crystals. Physiologically, calcification is restricted to bones and teeth by these factors. Similar mechanisms of calcification might be operative in extraosseous tissue, with inhibitory or promoting effects on local calcification [5]. Since the long-term outcome of patients with calcification is significantly impaired, identification of the factors and pathways that determine allograft calcification is necessary. Therefore, the aim of the present study was to analyze the contribution of the anti-calcifying factors matrix-Gla protein (MGP), osteopontin (OPN) and fetuin in biopsy samples as well as in urine and serum.

## Patients and Methods

The study was confirmed by the Ethical Committee of the Hannover Medical School and all patients gave their written informed consent. At our transplant centre, renal protocol biopsies are regularly performed at 6 weeks, 3 and 6 months after kidney or combined kidney/pancreas transplantation since 2001. Midstream spot-urine samples are collected immediately before the biopsy and are subsequently frozen at −80°C. Fresh urine samples are routinely analyzed for protein concentration and screened for hematuria and leukocyturia by dipstick analysis and microscopic inspection. Creatinine clearance was assessed using the Cockcroft-Gault equation. In addition, plasma and serum samples are collected at the indicated time points. From patients participating in this program, demographic and clinical data were collected from the time before and at the time of transplantation. Clinical data and routine laboratory results after transplantation were collected corresponding to the time points of the three protocol biopsies and 1-year post transplantation. All data were entered into a customized database (Oracle Enterprises, version 8.0.5).

From the available patient files in the database, 27 transplant patients without signs of graft calcification in any of the performed biopsies until 6 months (control) and 31 transplant patients with calcification (NC) were selected. To identify factors for calcification apart from serum parathormone and calcium, control patients were matched for serum calcium and parathyroid hormone levels at 6 weeks after transplantation but were otherwise randomly selected. Extensive comparisons between the selected patients and the whole group of patients in the database with regard to demographic data and those variables that were used for the subsequent analyses confirmed that the selected patients were representative of the whole group.

Protocol biopsies were evaluated according to the updated BANFF classification. Acute tubular injury was assessed according to the criteria described previously [6]. Besides routine stainings, *von Kossa* stain was performed on cases, which showed tubular or interstitial crystalloid deposits. The deposits were classified according to their localization in the luminal or tubulointerstitial area. Routine evaluation of the allograft biopsy includes examination of 18 biopsy sections, so that the possible risk of missing relevant calcification is low. Vascular calcifications were not present in any of the biopsies. Serum values of creatinine, calcium, phosphate and alkaline phosphatase activity were measured with an autoanalyzer. Parathyroid hormone levels were analyzed as the 7–84 fragment with a commercial kit. Vitamin D levels (1,25-dihydroxy Vitamin D) were assessed by a commercially available radioimmunoassay (Immunodiagnostic Systems, Germany).

### Immunohistochemistry

Immunohistochemistry for osteopontin, fetuin A and matrix-Gla protein was performed on formalin-fixed and paraffin-embedded tissue. For epitope retrieval serial sections of 5 µM were pre-treated by heat (microwave cooking, 30 min, pH 7.2). Polyclonal rabbit antisera against matrix-Gla protein (1∶50) and fetuin A (1∶50) (both from Atlas Antibodies, #HPA013949 and #HPA001524, Stockholm, Sweden) as well as osteopontin (1∶50) (Abcam, Cambridge, U.K.) were incubated for 1 h at room temperature and detected by a tyramine-amplified, avidin-biotin-based protocol [7], [8]. For evaluation the stained slides were separately examined for the presence (i.e. positive, including faint signals) or absence (i.e. negative) of the antigens. Negative controls for all antibodies were done by omitting the primary antibody and incubating with buffer instead.

### RNA Extraction and Quantitative Real-time PCR Analysis

Frozen biopsy samples were homogenized in an RLT-lysis buffer (Qiagen, Hilden, Germany), using a motor-driven homogenizer. Total RNA was extracted using RNeasy minicolumns with an on-column DNase digestion according to the manufacturer’s protocol (Qiagen). For real-time quantitative PCR, 2 µg total RNA was subjected to reverse transcription using a mix of random hexamers and oligo(dT) oligonucleotides. Quantitative reverse transcription–polymerase chain reaction analysis (qRT-PCR) was performed on an ICycler (Bio-Rad) with specific primers for osteopontin, fetuin A and matrix-Gla protein (Qiagen). Results were normalized to GAPDH levels as a housekeeping control.

### Analysis of Circulating and Urinary Antigens

Serum levels of MGP (Alpco Diagnostics, Salem, NH, U.S.A) and fetuin A (Biovendor Laboratory Medicine, Candler, NC, U.S.A.) as well as urinary levels of OPN (RnD Systems, Minneapolis, MN, U.S.A.) were analyzed by Enzyme-linked immunosorbent assay.

### Statistics

For the statistical analyses, the SPSS statistical software package, version 18.0.1 (SPSS Inc., Chicago, Illinois, USA), was used. Comparisons of categorical data between the groups and different biopsy time points were performed with the chi-square test for two or more samples. Numerical data were compared with the *t*-test and one-way ANOVA or with the *u*-test and Kruskal–Wallis test, dependent on the results of the analysis for normal distribution. Correlation analyses were performed using the Spearman rank test. Mean values are given with standard deviation, unless otherwise stated. Differences with p*<*0.05 were considered as being statistically significant.

## Results

### Patient Characteristics

**Figure 1:**
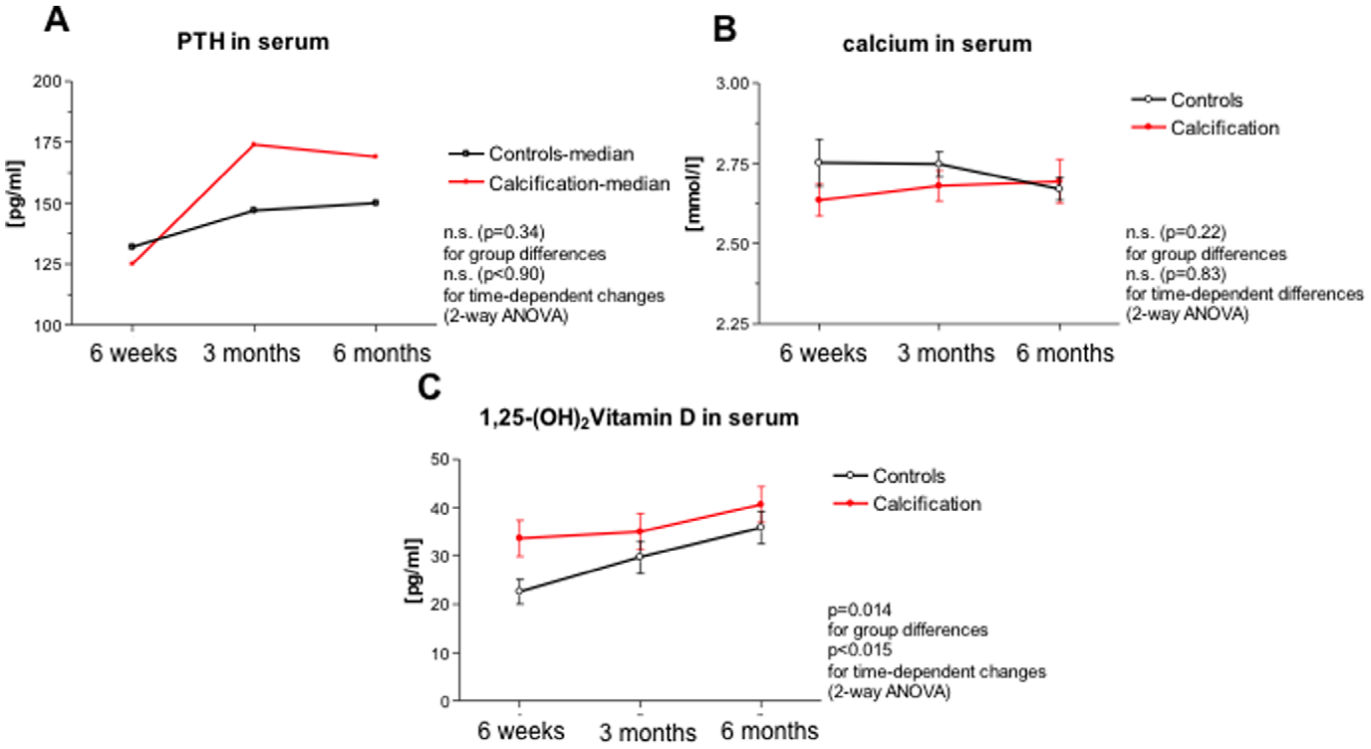
Levels of parathyroid hormone (A), serum calcium (B) and vitamin D (C) are shown in NC patients and controls at the indicated time points. Patients with calcification are depicted in red. Data are shown as mean ± standard error of the mean (SEM).

We included 27 transplant patients without signs of graft calcification (control) and 31 transplant patients with calcification (NC) that were matched for serum calcium and parathyroid hormone levels. Patient demographical data are displayed in **Table 1** **and** **2**. Parathyroid hormone, serum calcium and Vitamin D levels in NC patients and controls are shown in **Figure 1**.

**Table 1:** Demographics and transplant data of patients with and without (controls) calcification.

		Control	Calcification
Recipient gender Recipient age	*male/female years*	23/4 54.0±12.2	16/15* 50.0±12.1
Cause of ESRD			
Glomerulonephritis	*n*	8	8
Tubulointerstitial disease	*n*	2	5
Hypertensive/diabetic nephropathy	*n*	1	2
Other/unknown	*n*	3/13	5/11
Renal replacement therapy			
Hemodialysis	*n*	25	28
Peritoneal dialysis	*n*	2	2
None (pre-emptive Tx)	*n*	–	1
Time on dialysis	*Months*	72.5±38.3	69.0±36.0
PTH >150 ng/ml before Tx	*N*	14	18
Parathyroidectomy before Tx	*n*	4	6
Coronary heart disease	*n*	7	2
Peripheral artery disease	*n*	4	4
Previous kidney Tx	*1^st^/2^nd^ transplant*	25/2	28/3
Combined pancreas/kidney tx	*n*	1	-
Donor gender	*male/female*	15/12	18/13
Donor age	*Years*	53.6±14.1	50.7±17.2
Donor’s serum creatinine	*µmol/l*	80±24	83±40
Deceased/living kidney donation	*N*	22/5	25/6
HLA-mismatches			
locus A	*0/1/2*	9/10/8	20/6/5
locus B	*0/1/2*	8/13/6	10/13/8
locus DR	*0/1/2*	8/17/2	11/13/7
Sum of HLA-mismatches	*n*	2.7±1.7	2.3±1.9
Pre-formed PRA>0%	*n*	23	29
Initial immunsuppressive therapy			
Antibody induction	*ATG/IL-2*	2/25	1/21*
Cyclosporine A	*n*	18	16
Tacrolimus	*n*	6	14
Mycophenolate mofetil	*n*	23	23
Sirolimus/Evrolimus	*n*	2	1
Prednisolone	*n*	25	30
Delayed graft function	*n*	3	7
Cold ischemia time	*Hrs*	12.9±7.8	11.1±5.6
Lowest serum creatinine within the first 6 weeks post-Tx	*µmol/l*	138±54	133±51
Systolic blood pressure (median)	*mmHg*	125	128
Diastolic blood pressure (median)	*mmHg*	75	78

Continuous data are given as mean±SD unless otherwise stated;

* p = 0.011.

**Table 2:** Drug treatment and allograft status of patients and controls at biopsy time points.

	6 weeks		3 months		6 months	
	control	calcification	control	calcification	control	calcification
ARB’s/ACE-I’s§	62	38	63	32*	70	39*
Vitamin D suppl.	22	22	19	29	22	27
Phosphate suppl.	4	2	4	3	3	2
Cinacalcet	0	2	0	2	2	3
Hydronephrosis$	7	13	4	23+	19	13
Acute tubular injury	25	45*	30	30	33	45
cGrade&	15	16	7	32*	22	36
Serum creatinine *µmol/l (median; min-max))*	143 (63–298)	150 (65–276)	157 (70–1714)	152 (76–339)	153 (85–298)	152 (74–279)
Creatinine clearance *(ml/min/m^2^) (median; min-max))*	56 (27–111)	52 (18–93)	49 (4–123)	54 (14–114)	53 (24–97)	58 (21–103)

Values represent percentages.

* p<0.05;

+ p = 0.056.

§ ARB’s: angiotensin receptor blockers; ACE-I’s: Angiotensin converting enzyme inhibitors,

$ any grade of hydronephrosis,

& any cGrade>0 according to the BANFF classification.

There were no differences in patient and donor age. Time on dialysis and dialysis modality was similar. The number of female patients was significantly higher in the calcification group (p = 0.01, Table 1). The proportion of pre-existing hyperparathyroidism and rates of pre-transplant parathyroidectomy were comparable. Pre-transplant coronary heart disease was reported in seven patients without allograft calcification and in two patients with allograft calcification. Peripheral arterial disease of the lower limbs was present in three control patients and in four patients with allograft calcification.

Three control (11.1%) and 7 NC patients (22.5%) had delayed graft function which was defined as urine output of less than 500 ml in the first 24 h after transplantation and/or need of dialysis because of graft dysfunction within the first week after transplantation.

There was no difference with regard to the lowest serum creatinine within the first 6 weeks post transplantation. Moreover, patient groups did not differ concerning Vitamin D supplementation or phosphate supplementation. A few patients received cinacalcet at 6 weeks and 3 months after transplantation (NC: n = 2; control: n = 0) and 6 months after transplantation (NC: n = 2; control: n = 2). Coumarin was not given in any patient. The rate of hydronephrosis reached borderline significance in NC patients at 3 months after transplantation (p = 0.05). In addition, the proportion of interstitial fibrosis and tubular atrophy (cGrade >0 according to the BANFF classification) was higher in NC patients at 3 months after transplantation (p<0.05). At 6 weeks after transplantation, the number of NC patients with acute tubular necrosis was greater (p<0.05).

### Soluble Regulators of Calcification: Fetuin-A, MGP, OPN

**Figure 2:**
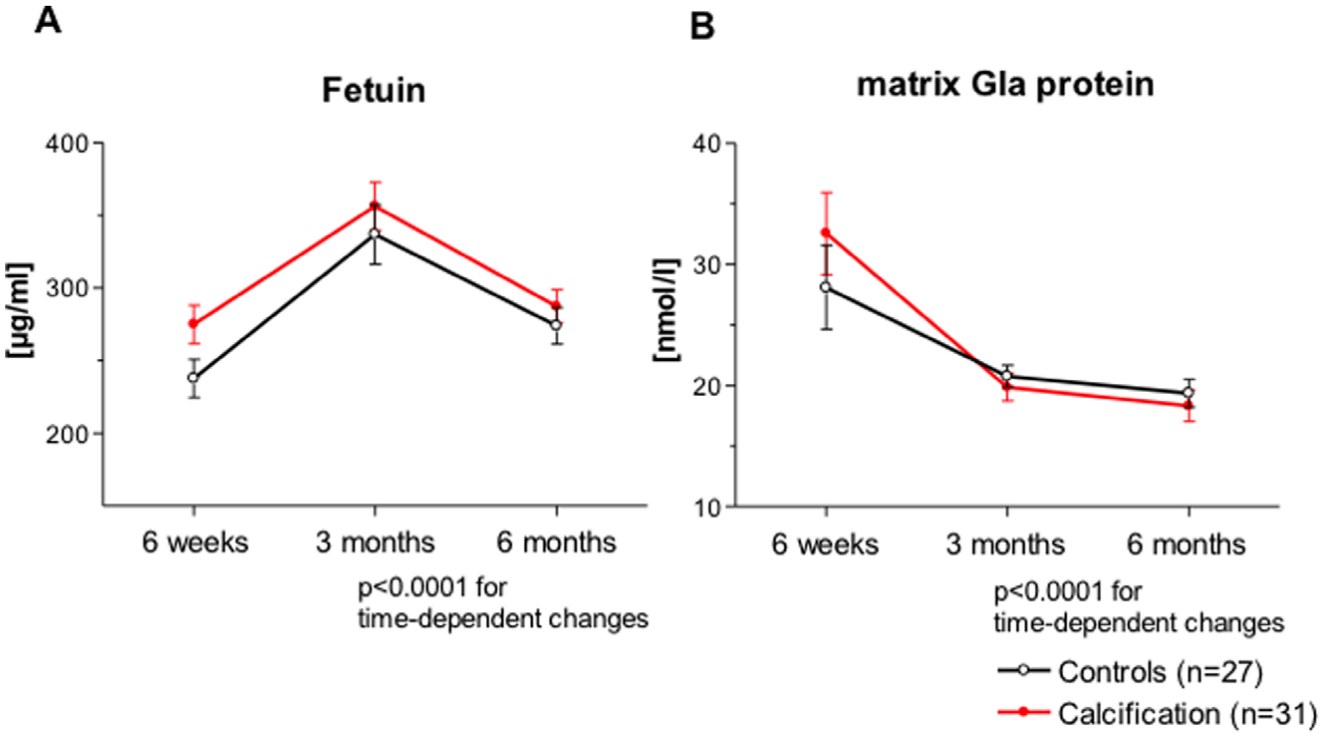
Serum concentrations of Fetuin A (A) as well as MGP (B) are shown in NC patients (n = 31) in comparison to control transplant patients without signs of calcification (n = 27) at the indicated time points. Patients with calcification are depicted in red.

**Figure 3:**
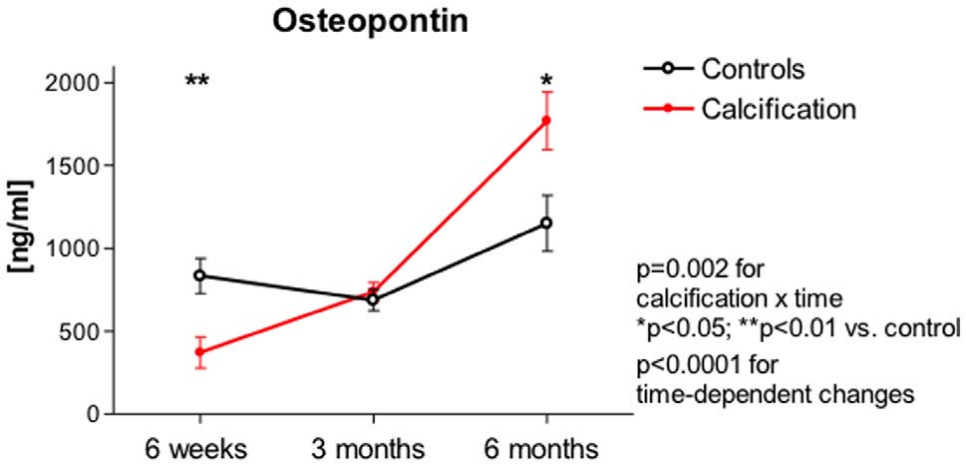
Urinary concentrations of OPN are shown in NC patients and control transplant patients without signs of calcification at the indicated time points. Patients with calcification are depicted in red.

Serum levels of fetuin-A and MGP as well as urinary levels of OPN showed highly significant time dependent changes in the first six months after transplantation that were not different between patients with and without calcification (**Fig. 2** **and** **3**). Urinary levels of OPN were different between patients with and without calcification (**Fig. 3** **)**. Levels of OPN were lower in NC patients at 6 weeks after transplantation compared to controls (409±482 ng/ml vs. 807±577 ng/ml; p<0.01) and increased at 3 months to levels that were comparable to those of the controls. At six months OPN levels in NC patients were significantly higher than in controls (1665.6±904.5 ng/ml vs. 1253.3±911.5 ng/ml; p<0.05). Serum levels of matrix-Gla protein did not differ between NC patients and controls at 6 weeks (32.6±18.9 ng/ml vs. 28.1±18 ng/ml), 3 months (19.9±6.4 ng/ml vs. 20.8±4.6 ng/ml) and 6 months after transplantation (18.3±7.3 ng/ml vs. 19.4±6.1 ng/ml). Similarly, a difference between NC patients and controls could not be detected concerning serum levels of fetuin (6 weeks: 275.1±73.3 ng/ml vs. 237.8±68.4 ng/ml; 3 months: 356.4±91.8 ng/ml vs. 337±105.7 ng/ml; 6 months: 287.5±63.9 ng/ml vs. 274.1±66.1 ng/ml).

### Tissue Expression of Antigens

The mRNA expression of MGP, fetuin and OPN in biopsy samples was assessed by quantitative real-time PCR. None of the antigens differed significantly in patients with and without calcification (MGP: p = 0.8; fetuin-A: p = 0.6; OPN: p = 0.4).

### Immunohistochemistry

**Figure 4:**
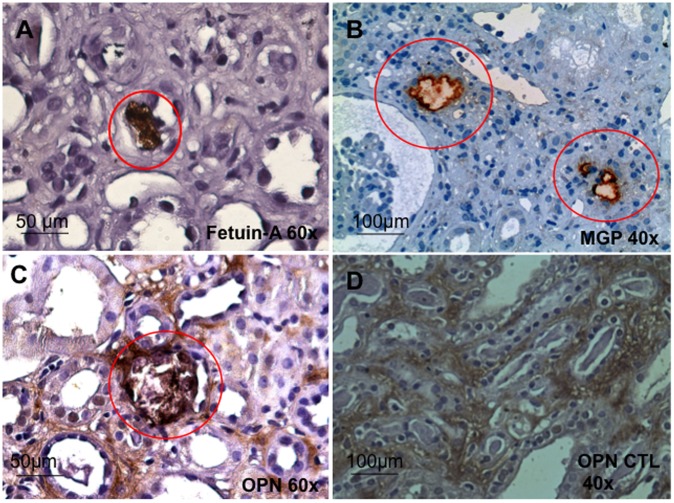
Representative images of Fetuin A (A), MGP (B) and OPN (C) expression are shown (red circle). The expression of the antigens is specifically localized in the vicinity of calcified plaques in patients with calcification. A representative control image is shown for Osteopontin (D). Original magnification: (Fetuin a and MGP x40; OPN x40 and x60).

The local expression of the anti-calcifying factors fetuin, OPN and MGP were specifically increased at the luminal surface of tubular epithelial cells around calcified plaques in NC patients (**Fig. 4**). Specifically, fetuin-A and MGP expression could be detected in the vicinity of tubulointerstitial calcifications, surrounding the deposits. OPN is specifically increased around calcified plaques. OPN could also be detected in tubular epithelial cells and interstitial cells in the control patients (see Figure 4D).

### Electrolytes in Patients with Calcification

**Figure 5:**
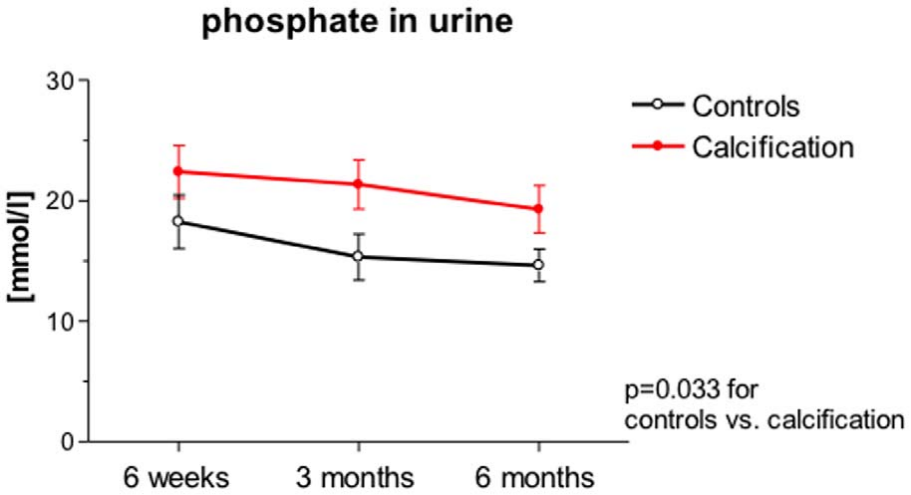
Urinary phosphate levels in NC patients in comparison to control transplant patients without signs of calcification at the indicated time points are shown. Patients with calcification are depicted in red.

We analyzed serum levels of calcium and phosphate as well as urine levels of phosphate, calcium, sodium, potassium, magnesium and uric acid. Mineral ions in urine are shown in **Table 3**. Phosphate concentrations in urine appeared to decline in both groups over time, with significantly higher phosphate levels in NC patients at the examined time points (p = 0.03, **Fig. 5**). Phosphate excretion was also determined relative to the creatinine excretion (i.e. phosphate:creatinine ratio), essentially yielding the same result of higher phosphate excretion in patients with calcification (not shown). We did not find differences in the remaining ions, neither in serum nor in urine.

**Table 3:** Urinary mineral ions in patients and controls at biopsy time points.

	6 weeks		3 months		6 months	
	control	calcification	control	calcification	control	calcification
Calcium (mmol/l) *(median; min-max)*	1.5 (0.1–6)	1.25 (0–5)	1.2 (0.1–3.3)	1.4 (0.1–6)	1.4 (0.1–6.7)	0.8 (0–6)
Phosphate (mmol/l) *(median; min-max)*	16.3 (5.8–60)	18.5 (4–49)	12.7 (2.6–48)	21 (4.6–53.5)	15 (5–29)	18 (0.7–39)
Sodium (mmol/l) *(median; min-max)*	105 (40–207)	84 (37–215)	87 (27–225)	85 (15–196)	91 (29–209)	83 (24–161)
Potassium (mmol/l) *(median; min-max)*	19.9 (9.7–53)	18 (9–59)	22.5 (7–65)	21 (10–47)	23 (4–59)	23 (7–66)
Magnesium (mmol/l) *(median; min-max)*	2 (0.3–6)	1.4 (0.3–5.4)	1.9 (0.5–6.5)	2.1 (0.3–6.3)	1.8 (0.7–6)	1.7 (0.4–4)

## Discussion

The aim of our present study was to further elucidate the mechanisms underlying calcification of renal allografts after we were previously able to demonstrate that patients with calcification have an inferior long-term graft outcome [4]. In our previous report, allograft calcification was merely associated with higher serum parathyroid hormone and calcium levels. Yet, the high overlap of these levels between NC patients and controls suggested that other factors must be operative in the induction of calcification. In order to identify these factors, we examined patient groups who had comparable parathyroid hormone and serum calcium levels. Patients groups were also comparable with regard to pre-existing hyperparathyroidism, length of dialysis treatment, initial graft function, and post-transplant therapies with vitamin D, phosphate supplements and cinacalcet. Post-transplant levels of 1,25-dihydroxy vitamin D were slightly higher in patients with calcification. The degree of acute tubular injury at 6 weeks after transplantation and the number of patients with hydronephrosis at 3 months after transplantation were slightly higher in patients with calcifications (see Table 2) suggesting that tissue injury may be a contributing factor in some cases with calcification.

Fetuin-A, MGP, and OPN have been reported as modulators of calcification in previous studies. These factors are present in body fluids and locally present in osseous and extra-osseous tissue and are believed to play a pivotal role in regulating calcium crystal deposition in bone and teeth and preventing unwanted calcification in extra-osseous tissue such as renal cells. [9]–[11]. Besides these factors, serum and urine ions which may be relevant for calcium crystal deposition [12] were analyzed in our study.

The results are as follows: (1) serum levels of fetuin and MGP as well as urinary levels of OPN have significant time dependent changes in the early course after transplantation. (2) Only urinary levels of OPN are different between patients with and without calcification. (3) Expression of these modulators is not different in biopsy specimens at the mRNA level. (4) The local protein expression of fetuin-A, MGP and OPN is specifically increased around calcified plaques. (5) The urinary excretion of phosphate is higher in patients with calcification.

Lack of OPN expression has been linked to increased extraosseous calcification as illustrated in OPN knock-out mice, which show increased vascular calcification [13]. Putative mechanisms of OPN actions include stimulation of phagocytic cells to clear calcified remnants, and induction of carbonic anhydrase II, which regulates local pH and mineral ion activity, physicochemical factors that are important for local precipitation of calcium-containing crystals and regression of mineral deposits [14]. In the kidney, OPN can directly prevent binding of calcium-containing crystals to renal epithelial cells [15], [16]. OPN is an important cardiovascular risk factor in patients with kidney disease [17], [18]. OPN is also secreted in the urine [19] and urinary levels of OPN have been suggested to reflect the local regulation of calcification in renal tissue [20]. In patients with urinary calculi, OPN secretion in urine was found to be reduced [21].We therefore investigated OPN levels in urine. On the other hand, increased local expression of OPN has also been linked to calcium crystal deposition. In rats with tubular damage induced by ethylene glycol, increased expression of OPN and hyaluronan in tubular epithelial cells was shown to be related to subsequent calcification at these sites [9]. In renal transplant patients the luminal expression of OPN and hyaluronan preceded retention of calcium-containing crystals at the distal tubule [22]. Similarly, we found OPN highly concentrated in the vicinity of calcium deposits. In the urine of patients with calcification, OPN was significantly decreased at six weeks after transplantation. Potential explanations include decreased renal expression of OPN (although we did not find differences in mRNA expression in the biopsies) or increased retention of OPN together with calcium crystals at the sites of tubulointerstitial calcification leading to lower OPN in urine. Thus, our patients with calcification might be characterized by a deficiency in OPN early after transplantation, which, among other factors, might be the basis for development of the calcifications. At later time points abundance of OPN is increased which most likely represents a local enrichment in response to the calcification.

For the first time, we report distinct time-dependent changes of serum fetuin and MGP in the early post-transplant course, pointing to an important role of these compounds in the regulation of post-transplant mineral metabolism. Fetuin-A may be viewed as chaperone for calcium ions. It forms a colloid with calcium and phosphate and is presumed to be cleared from the circulation by the reticuloendothelial system thus preventing unwanted calcium crystal deposition in extraosseous tissues [5]. In addition, fetuin-A is found in high concentrations in bone where it may be important for the local regulation of mineralization [10]. MGP functions as a vitamin K dependent regulator of local calcification [23], [24]. Genetic polymorphisms may influence the ability of matrix Gla protein to bind calcium-containing crystals, which has been linked to increased vascular calcification [25] and formation of urinary stones [26]. Matrix Gla protein is highly expressed in renal tubular epithelia [11].

Low serum levels of fetuin-A have been reported in patients with chronic renal failure which was associated with an increased rate of extraosseous calcification [27], [28]. Patients with a renal allograft were shown to have higher fetuin A and MGP levels and less vascular calcification compared to patients on dialysis [29].

Despite the remarkable changes of fetuin-A and MGP in the early post-transplant course, calcification was not associated with altered serum levels and renal mRNA expression of these proteins. Fetuin-A and MGP were found to be highly concentrated around the calcified plaques, seemingly encapsulating the calcium crystal deposits. It remains to be determined if this represents shedding of fetuin-A/MGP containing calcium complexes from the circulation or localized increased expression of these proteins in the tubulointerstitium which was not detected by the whole mRNA analysis of biopsies.

Lastly, we identified increased urinary phosphate excretion as a putative factor in the observed calcifications. Urinary phosphate concentration was approximately 25% higher at all three time points in patients with calcification. Increased phosphate excretion in the early post-transplant course is frequent and has been related to an impaired tubular reabsorption function due to tubular injury (immunosuppressants, ischemia/reperfusion) or increased presence of phosphatonins like FGF23 (fibroblast growth factor-23) [30]. Several other studies in non-transplanted patients have examined the effect of urinary excretion of calcium, phosphate and other ions demonstrating higher prevalences of nephrocalcinosis and stone formation in patients with higher daily amounts of these components [12], [31]. These studies also indicated that urine volume and the concentration of the different ions are more important compared to the absolute daily amounts as the ion concentrations determine supersaturation of the stone-forming components. As 24-hour determinations were not available in our patients we believe that the ion concentrations are best suited to reflect the disposition for calcium-phosphate precipitation. We also calculated urinary ion concentrations relative to urinary creatinine and basically observed the same results (not shown). Yet, there are no convincing data to show that ion concentrations relative to urinary creatinine reliably predict calcium phosphate precipitation. Unfortunately, plasma samples for FGF23 determination (FGF23 is detected in plasma) were only available in 21 patients with calcification and 17 without. In these patients, FGF23 levels were not different between those with and without calcification (not shown).

Our study has important limitations: The size of our patient cohort is small and represents a single-center experience. We cannot exclude with absolute certainty that a control patient had calcification in one or more biopsies due to the focal nature of the calcification findings (sampling error bias). However, routine evaluation of the allograft biopsy includes examination of 18 biopsy sections, so that the possible risk of missing relevant calcification is low. Differences in post-translational modification of MGP leading to activation were not evaluated in patients with and without calcification. Lastly, because of to the small sample size a multivariate analysis could not be performed to elucidate the relative contribution of the identified factors.

In conclusion, we identify differential osteopontin expression and phosphate excretion as putative factors of allograft calcification. Future studies have to analyze the involvement of circulating modulators and local tissue factors in larger cohorts of patients to further elucidate their importance in the prevention and resolution of calcification.
